# Crystal structure and chemistry of tricadmium digermanium tetra­arsenide, Cd_3_Ge_2_As_4_

**DOI:** 10.1107/S2056989019010636

**Published:** 2019-08-02

**Authors:** Michael R. Thompson, Brian J. Riley, Mark E. Bowden, Matthew J. Olszta, Danny J. Edwards, Jarrod V. Crum, Bradley R. Johnson, Saehwa Chong

**Affiliations:** a Pacific Northwest National Laboratory, Richland, WA 99354, USA

**Keywords:** cadmium germanium arsenide, crystal structure, XRD, EBSD

## Abstract

A cadmium germanium arsenide compound, Cd_3_Ge_2_As_4_, was synthesized using a double-containment fused quartz ampoule method within a rocking furnace and a melt-quench technique. The crystal structure was determined from single-crystal X-ray diffraction (SC-XRD), scanning and transmission electron microscopies (*i.e.* SEM, STEM, and TEM), and selected area diffraction (SAD) and confirmed with electron backscatter diffraction (EBSD). The chemistry was verified with electron energy loss spectroscopy (EELS).

## Abstract 2

A cadmium germanium arsenide compound, Cd_3_Ge_2_As_4_, was synthesized using a double-containment fused quartz ampoule method within a rocking furnace and a melt-quench technique. The crystal structure was determined from single-crystal X-ray diffraction, scanning and transmission electron microscopies, and selected area diffraction and confirmed with electron backscatter diffraction. The chemistry was verified with electron energy loss spectroscopy.

## Chemical context   

Crystalline *A* ^II^ *B* ^IV^ *C*_2_^V^ chalcopyrites such as CdGeAs_2_ are often studied for their non-linear optical properties (Boyd *et al.*, 1972 ▸; Byer, 1975 ▸; Byer *et al.*, 1971 ▸) amongst other promising applications. However, growing these materials in large volumes has proven difficult because of anisotropic thermal expansion that occurs during cooling (Iseler *et al.*, 1978 ▸; Boyd *et al.*, 1972 ▸; Schunemann & Pollak, 1998 ▸; Shay & Wernick, 1975 ▸; Kildal, 1972 ▸). During phase-diagram studies (Borshchevskii *et al.*, 1967 ▸; Borshchevskii & Roenkov, 1969 ▸) as well as crystal growth and syntheses efforts to produce amorphous Cd–Ge–As compounds (Hong *et al.*, 1990 ▸; Mikkelsen Jr & Hong, 1974 ▸; Pamplin & Feigelson, 1979 ▸; Sharma *et al.*, 1989 ▸; Zawilski *et al.*, 2008 ▸), several different Cd–Ge–As impurity phases were observed in addition to the target CdGeAs_2_ compound. These include Cd_7_Ge_91_As_2_ (Hong *et al.*, 1990 ▸), Cd_13_Ge_81_As_6_ (Hong *et al.*, 1990 ▸), Cd_28_Ge_7_As_65_ (Hong *et al.*, 1990 ▸), Cd_29_Ge_14_As_57_ (Mikkelsen Jr & Hong, 1974 ▸), Cd_33_Ge_11_As_56_ (Hong *et al.*, 1990 ▸), and Cd_33_Ge_25_As_42_ (Pamplin & Feigelson, 1979 ▸). During separate experiments by the authors where CdGeAs_2_ materials were being synthesized for amorphous radiation detectors (Johnson *et al.*, 2009 ▸), a phase with the composition of ∼Cd_33_Ge_22_As_45_ (or Cd_3_Ge_2_As_4_) was observed. This phase was not observed during any of the studies previously reported in the literature (Hong *et al.*, 1990 ▸; Mikkelsen Jr & Hong, 1974 ▸; Pamplin & Feigelson, 1979 ▸; Sharma *et al.*, 1989 ▸; Zawilski *et al.*, 2008 ▸; Schunemann & Pollak, 1998 ▸; Speyer *et al.*, 1989 ▸), and the formation of this crystalline phase is thought to be due to the unique double-containment ampoule method used during synthesis.

In our previous paper (Riley *et al.*, 2012 ▸), we described the synthesis process for maximizing the production of the Cd_3_Ge_2_As_4_ crystalline phase where the target composition was batched and processed under similar conditions used for making CdGeAs_2_, including the double-containment ampoule method. In this paper, we report the crystal structure of the Cd_3_Ge_2_As_4_ crystalline phase; using XRD and analytical electron microscopies, it was shown to belong to the trigonal system in the *R* 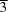 space group (No. 148). The SC-XRD analysis of small shards of the crystalline phase were utilized to propose the atomic positions of each of the elements, and subsequent use of a double-tilt stage holder in (S)TEM coupled with EELS analysis confirmed these data. Through the combination of these two techniques, the structure of this newly formed phase could be accurately described. Using the crystallographic information file (CIF) created with SC-XRD, EBSD was used to demonstrate successful pattern indexing from the Kikuchi patterns.

## Structural commentary   

**Figure 1:**
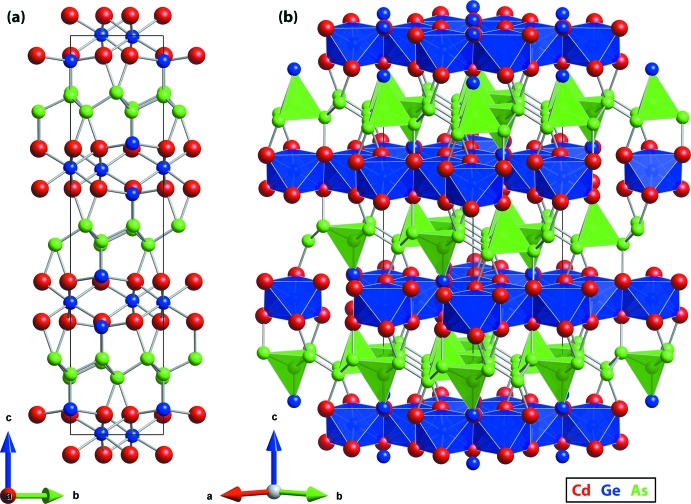
Crystal structure of the Cd_3_Ge_2_As_4_ showing (*a*) the distribution and linkages of atoms and (*b*) the layer structure composed of GeAs_4_ tetra­hedra and GeCd_6_ octa­hedra; the legend is shown in the bottom right (Cd = red, Ge = blue, As = green). The reader is referred to the online version for reference to color.

**Figure 2:**
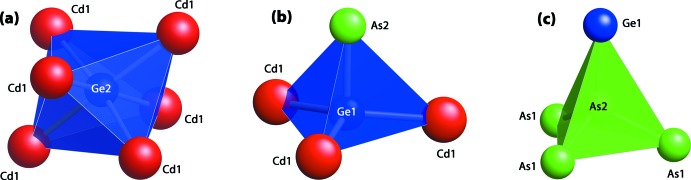
Coordinated atoms around Ge2, Ge1, and As2 are shown as (*a*) a Ge2(Cd1)_6_ octa­hedron, (*b*) a Ge1[(Cd1)_3_As2] tetra­hedron, and (*c*) an As2[(As1)_3_Ge1] tetra­hedron.

The SC-XRD data yielded a trigonal unit cell of space group *R* 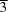 (No. 148) with lattice parameters *a*,*b* = 7.3748 (13) Å and *c* = 27.415 (5) Å (see Table 1 ▸). The heavy-scattering Cd atom was assigned to a general position *ca* 1.4 Å above the *ab* plane with some confidence, but Ge and As have similar scattering factors and were distributed amongst the remaining sites with less certainty. The lowest *R* factor (3.62% for all data) was found for the final refinement of the atomic poisitions, which also agreed with the expected stoichiometry of Cd_3_Ge_2_As_4_. The structure is shown in Fig. 1 ▸ and contains sheets of metal atoms stacked along the *c* axis. The Cd site and both As sites are tetra­hedrally coordinated to neighboring metal atoms. The two Ge sites have tetra­hedral (to three Cd and one As) and octa­hedral (to six Cd) coordination environments, respectively (see Fig. 2 ▸). Having Ge in these two environments is consistent with the expected valences of the metal atoms. If Cd and As are assigned their expected oxidation states of Cd^2+^ and As^3−^, respectively, then one Ge^2+^ and one Ge^4+^ are required for charge neutrality.

## Synthesis and characterization   

**Figure 3:**
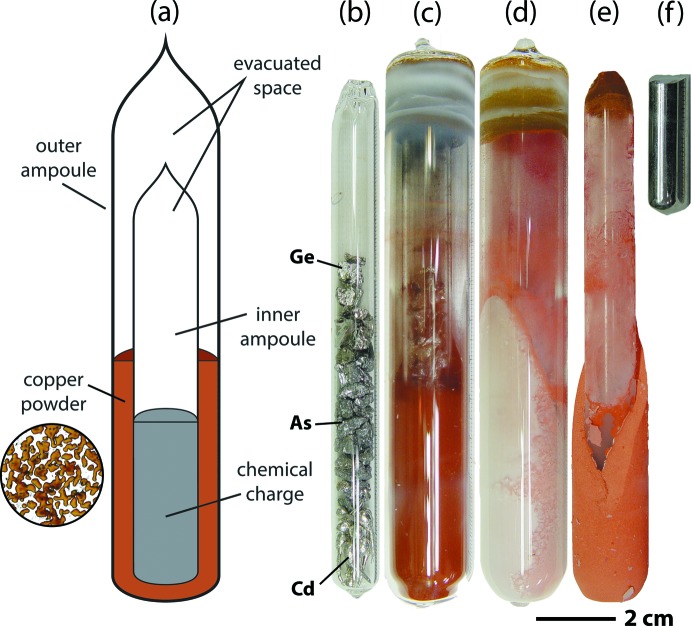
(*a*) Schematic of double-containment ampoule setup showing a high magnification of the sintered copper powder. (*b*–*f*) Progression of steps involved in the double-containment ampoule process [with scalebar for (*b*)–(*f*)] showing (*b*) elements packed in an evacuated and sealed 10×12 mm inner FQ tube, (*c*) the inner ampoule loaded into an evacuated and sealed outer 19×22 mm FQ tube containing copper powder, (*d*) the assembly after heat treatment, (*e*) the assembly removed from the outer ampoule, and (*f*) the ingot removed from the inner ampoule. This figure was recreated with permission from the American Ceramic Society.

The Cd_3_Ge_2_As_4_ sample, made using a double-containment quartz ampoule setup (see Fig. 3 ▸ *a*), is discussed elsewhere in more detail (Johnson *et al.*, 2009 ▸; Riley *et al.*, 2012 ▸), but will be briefly described here. For the double-containment ampoule setup, high purity Cd (4.4916 g, 99.9999%), Ge (2.9030 g, 99.9999%), and As (5.9907 g, 99.99999+%) (Alfa Aesar, Ward Hill, MA) were added into a pre-cleaned fused quartz vessel [10×12 mm; GE214, GM Associates, Inc., Oakland, CA; cleaning details are provided elsewhere (Riley *et al.*, 2012 ▸)] while inside a nitro­gen glovebox (< 0.1 ppm of O_2_/H_2_O; M-Braun, Inc., Stratham, NH). Then, the vessel was moved out of the glovebox while connected to a sealed gate valve and 12.5-mm compression fitting to make sure that the contents of the ampoule were not exposed to the atmosphere. Then, the ampoule was evacuated, purged with semiconductor grade Ar/2.6%H_2_ to 1.6×10^4^ Pa (120 Torr), and sealed with an oxy-propane torch. This is the *inner ampoule* shown in Fig. 3 ▸ *b*. This inner ampoule was loaded into a 19×22 mm fused quartz tube (*outer ampoule*, Fig. 3 ▸ *c*,*d*) that was sealed at the base, and the annulus between the two vessels was filled with 17.8 g of Cu powder (≤ 75 µm). The ampoule was heat treated in a rocking furnace where it was ramped at 3°C min^−1^ from room temperature to 400°C, held for 2 h in a fixed position, ramped to 625°C at 3°C min^−1^, held for 2 h, ramped to 850°C at 3°C min^−1^, held for 25 h, rocking was paused for 10 min to allow the melt to settle to the base, and then the ampoule assembly was rapidly removed from the furnace and quenched in an ice bath filled with NaCl.

Upon cooling, the ingot was cut into discs and polished in oil-based diamond suspensions for further analysis. Optical microscopy was utilized with cross-polarized light to visualize cross-sectioned specimens at various magnifications (12.5–1000×) using a Leitz Orthoplan optical microscope (Leica Microsystems GmbH, Wetzlar, Germany). Imaging was performed using a Sony DSC-F717 digital camera connected to the microscope through a C-mount adapter.

A crystal suitable for structure determination was identified amongst the small pieces, which broke off one of the cut discs. A series of Omega scans were collected at 150 K from an untwinned crystal 30×70×70 µm using a Bruker APEXII diffractometer (Bruker AXS Inc., Madison, WI) and Mo *K*_α_ radiation (more details are provided in the Refinement section). Absorption corrections were applied using the multi-scan method and the structure refined with *SHELXTL*.

The EBSD analysis was performed on a polished cross section of the sample using a Bruker e-Flash HR (Bruker AXS Inc., Madison, WI) coupled to a JSM-7001F field-emission gun SEM (JEOL USA, Inc., Peabody, MA). Mapping was performed under 30 kV acceleration voltage at 200× magnif­i­cation at 1024×768 pixel resolution (0.2789 mm^2^ map size), a 12.9 frames per second average collection rate, a 2.9° detector tilt angle, and a 70° sample tilt angle. Data were analyzed using ESPRIT (v1.9) software (Bruker AXS Inc.). Data processing revealed a 92.7% phase match with 7.3% of unidentified regions (zero solutions).

The STEM and TEM analyses were performed on an electron-transparent lamella (∼5×10 µm^2^) prepared *via* a cross-sectional lift-out technique using a FEI Quanta 3D focused ion beam (FIB). A JEOL ARM 200CF, a cold field emission, aberration probe corrected TEM (JEOL USA, Inc.; Peabody, MA) operated at 200 kV was utilized to examine these samples. Several techniques were used, including SAD in conventional TEM mode, STEM high-angle annular darkfield (HAADF) and annular brightfield (ABF) as well as EELS. The EELS analysis was performed on a Gatan Quantum 965 ER Gatan Image Filter (Gatan Inc., Pleasanton, CA) equipped with fast shutter and dual EELS modules. The EELS spectra and spectroscopic mapping were performed with a convergence angle of 27.5 mrad and a collection angle of 90 mrad using the Ge *L*_α_, As *L*_α_, and Cd *M*_α_ lines. Background subtraction and spectral math were performed with the Gatan Digital Microscopy Suite (v3.0).

## Refinement   

Crystal data, data collection and structure refinement details are summarized in Table 1 ▸. A suitable crystal for SC-XRD was selected and mounted on a Bruker APEXII diffractometer with a microfocus Mo-K_α_ X-ray tube and a CCD detector. Data were collected with 0.7° frame widths in ω and 20 s dwell times per frame with the crystal held at 150 K. A total of 2198 frames were collected, and the total exposure time was 12.21 h. The frames were integrated with the *SAINT* (Bruker, 2012 ▸) software package using a narrow-frame algorithm. The integration of the data using a trigonal unit cell yielded a total of 20756 reflections to a maximum θ angle of 42.35° (0.53 Å resolution), of which 2026 were independent (average redundancy 10.245, completeness = 98.6%, *R*_int_ = 6.18%, *R*_sig_ = 3.19%) and 1702 (84.01%) were greater than 2σ(|*F*|^2^). The final cell constants of *a* = 7.3748 (13) Å, *b* = 7.3748 (13) Å, *c* = 27.415 (5) Å, and volume = 1291.3 (5) Å^3^, are based upon the refinement of the *XYZ*-centroids of 6406 reflections above 20 σ(*I*) with 8.723° < 2θ < 82.60°. Data were corrected for absorption effects using the multi-scan method with the *SADABS* software program (Bruker, 2001 ▸). The ratio of minimum-to-maximum apparent transmission was 0.476. The calculated minimum and maximum transmission coefficients (based on crystal size) were 0.2330 and 0.4730. The structure was solved and refined using the *SHELXTL* (Sheldrick, 2008 ▸) software package, using the space group *R* 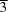, with *Z* = 6 for the formula unit, As_4_Cd_3_Ge_2_ (referred to here as Cd_3_Ge_2_As_4_). The final anisotropic full-matrix least-squares refinement on *F* ^2^ with 29 variables converged at *R*_1_ = 2.53%, for the observed data and w*R*_2_ = 4.91% for all data. The goodness-of-fit was 1.032. The largest peak in the final difference electron density synthesis was 1.996 e^−^ Å^−3^ and the largest hole was −2.180 e^−^ Å^−3^ with an r.m.s. deviation of 0.324 e^−^ Å^−3^. On the basis of the final model, the calculated density was 6.034 Mg m^−3^ and *F*(000), 2040 e^−^.

## Results and discussion   

**Figure 4:**
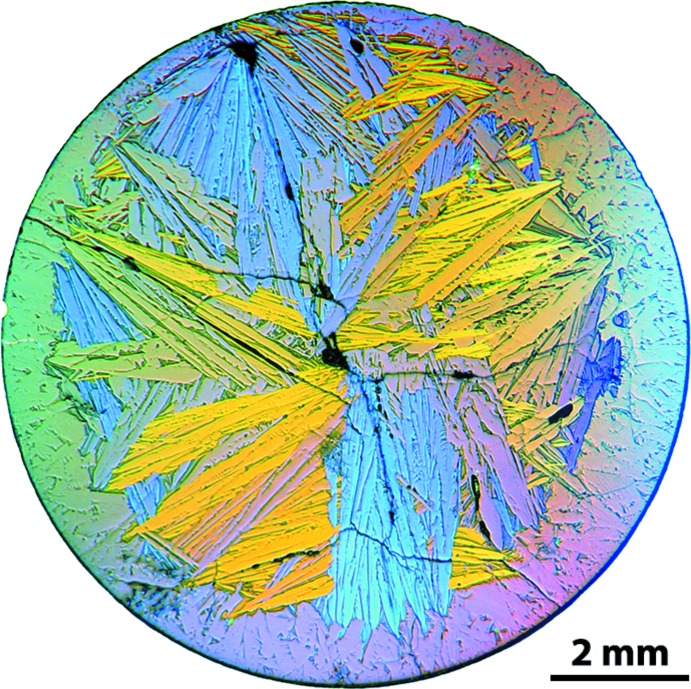
Cross-polarized light optical micrograph of a cross-sectioned sample disc after polishing showing the crystals within the core and the amorphous rim around the perimeter.

Through the progression of samples fabricated to optimize production of the Cd_3_Ge_2_As_4_ crystals (Riley *et al.*, 2012 ▸), optical microscopy was used to assess macroscopic features and crystal yield. While the crystals and the glassy matrix have similar compositions, the crystals could be separately visualized using cross-polarized light illumination; this provided a technique for broadly assessing the crystallite size. This can be seen in Fig. 4 ▸ where the crystallites populate the core of the disc and the amorphous outer layer can be separately visualized.

**Figure 5:**
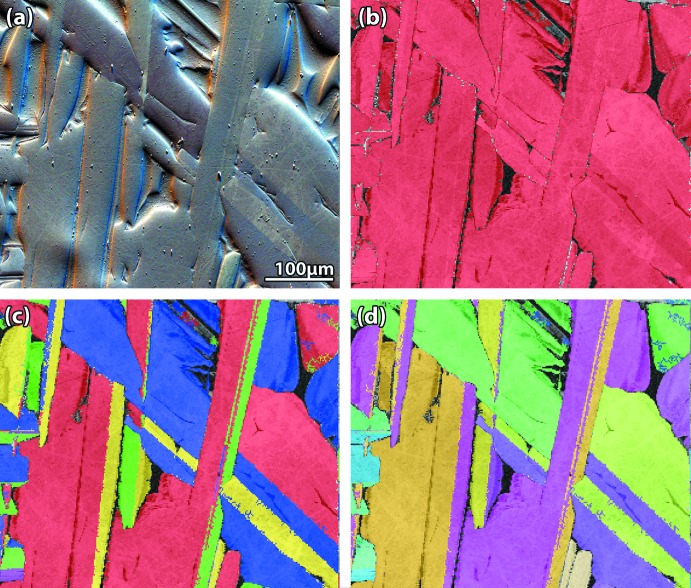
EBSD pattern collage including the (*a*) Argus image, (*b*) phase map refined using the crystallographic information file (CIF), (*c*) grain map, and (*d*) inverse pole figure (in the *X*-direction) map.

The EBSD mapping shown in Fig. 5 ▸ confirms that the CIF generated from SC-XRD analysis of the Cd_3_Ge_2_As_4_ crystal indexed well to the Kikuchi patterns. This data was used to corroborate the SC-XRD data. The orientation maps presented in Fig. 5 ▸ *c* demonstrate that the grain sizes can span several hundred micrometers.

**Figure 6:**
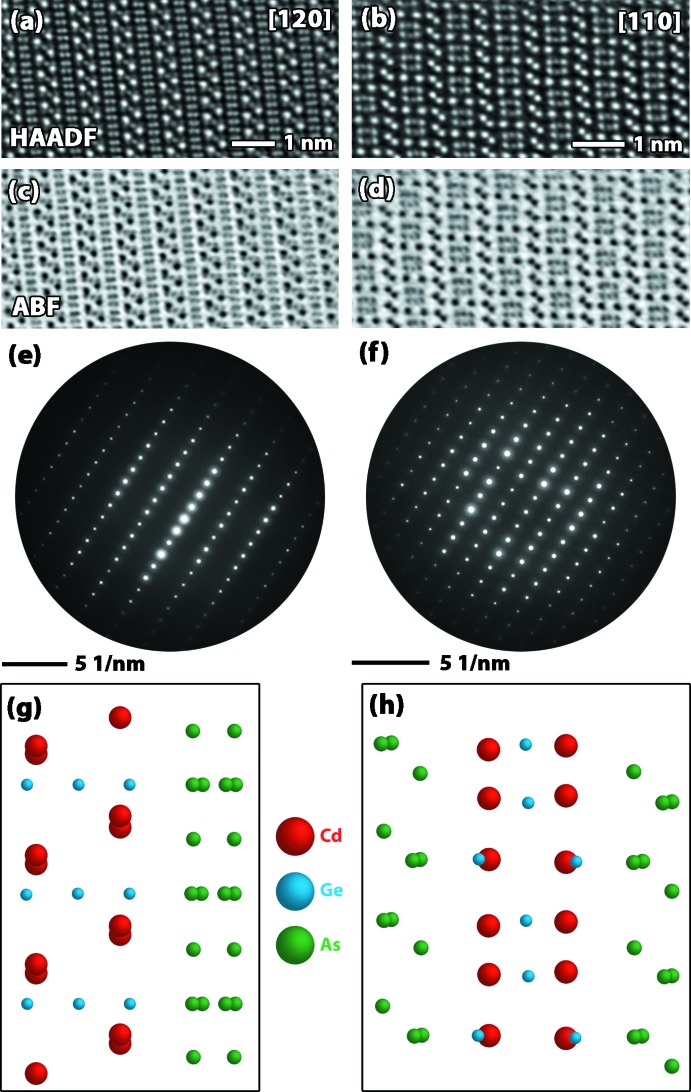
(*a*)–(*d*) Atomic-resolution column micrographs in STEM (*a*),(*b*) HAADF and (*c*),(*d*) ABF imaging modes as well as the (*e*),(*f*) selected area diffraction and (*g*),(*h*) corresponding atomic identifications for each pole (*i.e.*, [120] on the left and [110] on the right).

Utilizing both the diffraction patterns in TEM mode and the Kikuchi map in STEM Ronchigram mode, the sample was tilted to obtain low index zone axes in order to better understand the crystallography of the material. Fig. 6 ▸ shows HAADF, ABF, and diffraction patterns collected from the {11-20} and {21-30} type planes of the trigonal crystal. Ball-and-stick models of these orientations (derived from previously mentioned XRD studies) matched extremely well with the atomic column imaging collected in STEM mode. While slight drift in the imaging of the sample during data collection is apparent in the atomic column images, the local ordering could be related to the XRD data. The contrast of the HAADF images was used to corroborate the symmetry and the location of the atoms, but since the predicted structure did suggest overlap of atomic species, using the contrast in the HAADF and ABF images was insufficient to explicitly prove the structure.

**Figure 7:**
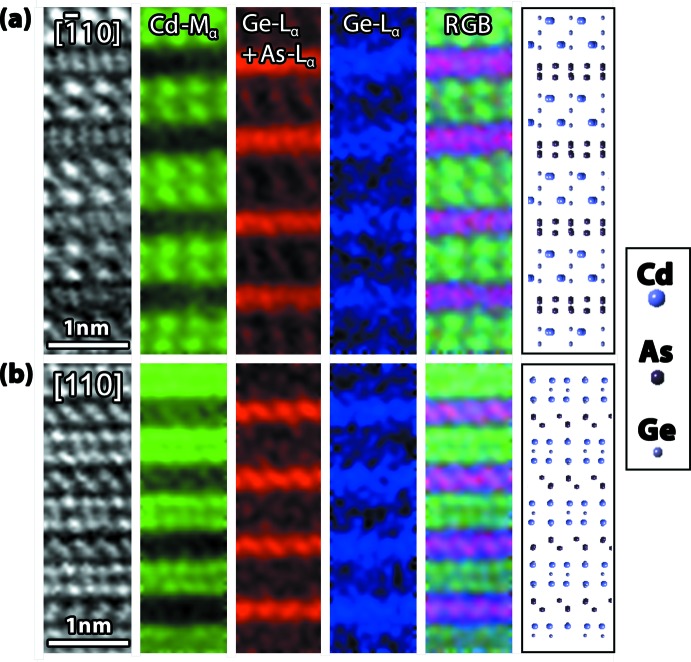
Atomic column EELS maps for two crystallographic poles, *i.e.*, (*a*) [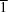
10] and (*b*) [110], in the trigonal system to different regions within the crystal (note these are rotated 90° from the images in Fig. 6 ▸).

**Figure 8:**
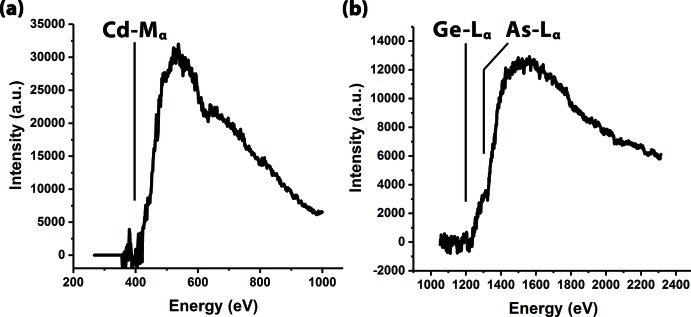
EELS spectra showing the identification of (*a*) Cd-*M*_α_, (*b*) Ge-*L*_α_, and (*b*) As-*L*_α_ energy lines that were used for elemental identification and mapping.

Atomic column dual EELS mapping shown in Fig. 7 ▸ provided a clearer picture of the chemical location of each of the atoms within the crystal. The Cd-*M*_α_ edge, starting at 403.7 eV (see Fig. 8 ▸), was utilized to map the location of the Cd elements in both orientations shown in Fig. 6 ▸. In the {12-30} orientation, the distinct Cd positions could be easily mapped without overlap from either the Ge or As. In the {11-20} orientation, the Cd overlapped with the Ge, and hence the signal was slightly obscured, but individual atomic positions could still be observed. The Ge and the As signals were more complicated because of their *L*_α_ edge onsets at 1217 and 1323 eV, respectively. The higher energy edges were more difficult to map because of shorter collection times and low signal-to-noise ratios. Additionally, since the edges are so close in energy, background subtraction and signal integration were difficult. Theoretically, the Ge-*L*_α_ edge could be decoupled from the As-*L*_α_ edge, but because of the minimal amount of integration before the As-*L*_α_ edge, the amount of intensity does not provide adequate information for successful deconvolution.

In EELS, the As-*L*_α_ signal will always be convoluted with the Ge-*L*_α_ signal, so exact identification of the As *versus* the Ge proved difficult. Mapping of the Ge-*L*_α_ edge before the As-*L*_α_ edge (∼1217–1320 eV) provided a map that suggests there are Ge and As substitutions in both orientations. When the As-*L*_α_ + Ge-*L*_α_ edges were utilized for mapping, the signal validated the XRD data in that there were individual layers of As in both orientations. Mapping of the Ge-*L*_α_ edge showed little Ge amongst the Cd layers, but when the Ge and As signals were used, the atomic positions suggested by XRD and HAADF were expressed. Again, since it is difficult to deconvolve the two signals, atomic column mapping could not be used to definitively identify the positions of the As and Ge atoms within the crystal structure. Difference maps are also provided in Fig. 7 ▸ (*i.e*., RGB mix), which give a good indication of the positions of the elements in relation to one another.

## Supplementary Material

Crystal structure: contains datablock(s) global, I. DOI: 10.1107/S2056989019010636/vn2150sup1.cif

Structure factors: contains datablock(s) I. DOI: 10.1107/S2056989019010636/vn2150Isup2.hkl

CCDC reference: 1943551

Additional supporting information:  crystallographic information; 3D view; checkCIF report

## Figures and Tables

**Table 1:** Experimental details

Crystal data	
Chemical formula	Cd_3_Ge_2_As_4_
*M*_r_	782.06
Crystal system, space group	Trigonal, *R* 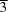
Temperature (K)	150
*a*, *c* (Å)	7.3748 (13), 27.415 (5)
*V* (Å^3^)	1291.3 (5)
*Z*	6
Radiation type	Mo *K*α
μ (mm^−1^)	29.36
Crystal size (mm)	0.07 × 0.07 × 0.03

Data collection	
Diffractometer	Bruker APEXII CCD area detector
Absorption correction	Multi-scan (*SADABS*; Bruker, 2001 ▸)
*T*_min_, *T*_max_	0.147, 0.414
No. of measured, independent and observed [*I* > 2σ(*I*)] reflections	20756, 2026, 1702
*R*_int_	0.062

Refinement	
*R*[*F* ^2^ > 2σ(*F* ^2^)], *wR*(*F* ^2^), *S*	0.025, 0.049, 1.03
No. of reflections	2026
No. of parameters	29
Δρ_max_, Δρ_min_ (e Å^−3^)	2.00, −2.18

Computer programs: *APEX2* and *SAINT* (Bruker, 2012 ▸), *SHELXTL* (Sheldrick, 2008 ▸), *VESTA* (Momma & Izumi, 2011 ▸) and *publCIF* (Westrip, 2010 ▸).

## supplementary crystallographic information

### Crystal data

**Table:**

Cd_3_Ge_2_As_4_	*D*_x_ = 6.034 Mg m^−^^3^
*M**_r_* = 782.06	Mo *K*α radiation, λ = 0.71073 Å
Trigonal, *R*3	Cell parameters from 20756 reflections
Hall symbol: -R 3	θ = 2.2–42.4°
*a* = 7.3748 (13) Å	µ = 29.36 mm^−^^1^
*c* = 27.415 (5) Å	*T* = 150 K
*V* = 1291.3 (5) Å^3^	Triangular prism, black
*Z* = 6	0.07 × 0.07 × 0.03 mm
*F*(000) = 2040	

### Data collection

**Table:**

Bruker APEXII CCD area detector diffractometer	1702 reflections with *I* > 2σ(*I*)
ω and φ scans	*R*_int_ = 0.062
Absorption correction: multi-scan (SADABS; Bruker, 2001)	θ_max_ = 42.4°, θ_min_ = 2.2°
*T*_min_ = 0.147, *T*_max_ = 0.414	*h* = −13→13
20756 measured reflections	*k* = −13→13
2026 independent reflections	*l* = −51→51

### Refinement

**Table:**

Refinement on *F*^2^	Primary atom site location: structure-invariant direct methods
Least-squares matrix: full	*w* = 1/[σ^2^(*F*_o_^2^) + (0.0102*P*)^2^ + 16.7635*P*] where *P* = (*F*_o_^2^ + 2*F*_c_^2^)/3
*R*[*F*^2^ > 2σ(*F*^2^)] = 0.025	(Δ/σ)_max_ = 0.001
*wR*(*F*^2^) = 0.049	Δρ_max_ = 2.00 e Å^−^^3^
*S* = 1.03	Δρ_min_ = −2.18 e Å^−^^3^
2026 reflections	Extinction correction: SHELXL2016 (Sheldrick, 2015), Fc^*^=kFc[1+0.001xFc^2^λ^3^/sin(2θ)]^-1/4^
29 parameters	Extinction coefficient: 0.00068 (3)
0 restraints	

### Special details

**Table:**

Geometry. All esds (except the esd in the dihedral angle between two l.s. planes) are estimated using the full covariance matrix. The cell esds are taken into account individually in the estimation of esds in distances, angles and torsion angles; correlations between esds in cell parameters are only used when they are defined by crystal symmetry. An approximate (isotropic) treatment of cell esds is used for estimating esds involving l.s. planes.
Refinement. Reflections were merged by SHELXL according to the crystal class for the calculation of statistics and refinement. _reflns_Friedel_fraction is defined as the number of unique Friedel pairs measured divided by the number that would be possible theoretically, ignoring centric projections and systematic absences.

### Fractional atomic coordinates and isotropic or equivalent isotropic displacement parameters (Å^2^)

**Table:**

	*x*	*y*	*z*	*U*_iso_*/*U*_eq_	
Cd1	0.63686 (3)	0.64317 (3)	0.05011 (2)	0.01073 (5)	
As1	0.49777 (4)	0.50674 (4)	0.14283 (2)	0.00577 (5)	
As2	1.000000	1.000000	0.15184 (2)	0.00553 (7)	
Ge1	1.000000	1.000000	0.06367 (2)	0.00604 (8)	
Ge2	0.666667	0.333333	0.00242 (2)	0.00535 (7)	

### Atomic displacement parameters (Å^2^)

**Table:**

	*U*^11^	*U*^22^	*U*^33^	*U*^12^	*U*^13^	*U*^23^
Cd1	0.01246 (8)	0.01108 (8)	0.00932 (7)	0.00638 (7)	−0.00329 (6)	−0.00271 (6)
As1	0.00541 (9)	0.00536 (10)	0.00664 (9)	0.00277 (8)	−0.00009 (7)	0.00023 (7)
As2	0.00523 (10)	0.00523 (10)	0.00615 (16)	0.00261 (5)	0.000	0.000
Ge1	0.00695 (11)	0.00695 (11)	0.00422 (16)	0.00348 (5)	0.000	0.000
Ge2	0.00442 (10)	0.00442 (10)	0.00722 (17)	0.00221 (5)	0.000	0.000

### Geometric parameters (Å, º)

**Table:**

Cd1—Ge1	2.6810 (5)	As1—As2^ii^	2.4296 (5)
Cd1—Ge2	2.7352 (5)	As1—As1^iii^	2.4496 (5)
Cd1—As1	2.7374 (5)	As1—As1^iv^	2.4496 (5)
Cd1—Ge2^i^	2.7390 (5)	As2—Ge1	2.4172 (8)
Cd1—Cd1^i^	3.4379 (6)		
			
Ge1—Cd1—Ge2	115.352 (9)	As2—Ge1—Cd1	97.973 (11)
Ge1—Cd1—As1	103.815 (15)	As2—Ge1—Cd1^vi^	97.973 (11)
Ge2—Cd1—As1	107.978 (14)	Cd1—Ge1—Cd1^vi^	118.108 (5)
Ge1—Cd1—Ge2^i^	117.239 (9)	As2—Ge1—Cd1^vii^	97.973 (11)
Ge2—Cd1—Ge2^i^	102.189 (11)	Cd1—Ge1—Cd1^vii^	118.109 (5)
As1—Cd1—Ge2^i^	110.095 (15)	Cd1^vi^—Ge1—Cd1^vii^	118.108 (5)
Ge1—Cd1—Cd1^i^	134.854 (15)	Cd1^viii^—Ge2—Cd1^ix^	99.051 (15)
Ge2—Cd1—Cd1^i^	51.144 (9)	Cd1^viii^—Ge2—Cd1	99.050 (14)
As1—Cd1—Cd1^i^	121.283 (16)	Cd1^ix^—Ge2—Cd1	99.050 (14)
Ge2^i^—Cd1—Cd1^i^	51.045 (9)	Cd1^viii^—Ge2—Cd1^x^	88.526 (12)
As2^ii^—As1—As1^iii^	97.897 (17)	Cd1^ix^—Ge2—Cd1^x^	77.810 (11)
As2^ii^—As1—As1^iv^	99.093 (17)	Cd1—Ge2—Cd1^x^	172.207 (10)
As1^iii^—As1—As1^iv^	94.181 (17)	Cd1^viii^—Ge2—Cd1^xi^	77.810 (11)
As2^ii^—As1—Cd1	113.706 (15)	Cd1^ix^—Ge2—Cd1^xi^	172.207 (10)
As1^iii^—As1—Cd1	140.455 (14)	Cd1—Ge2—Cd1^xi^	88.526 (11)
As1^iv^—As1—Cd1	103.154 (16)	Cd1^x^—Ge2—Cd1^xi^	94.898 (15)
Ge1—As2—As1^ii^	115.864 (13)	Cd1^viii^—Ge2—Cd1^i^	172.207 (10)
Ge1—As2—As1^v^	115.864 (13)	Cd1^ix^—Ge2—Cd1^i^	88.526 (11)
As1^ii^—As2—As1^v^	102.389 (15)	Cd1—Ge2—Cd1^i^	77.810 (11)
Ge1—As2—As1^iv^	115.864 (13)	Cd1^x^—Ge2—Cd1^i^	94.898 (15)
As1^ii^—As2—As1^iv^	102.389 (15)	Cd1^xi^—Ge2—Cd1^i^	94.898 (15)
As1^v^—As2—As1^iv^	102.388 (15)		

Symmetry codes: (i) −*x*+1, −*y*+1, −*z*; (ii) −*x*+5/3, −*y*+4/3, −*z*+1/3; (iii) *y*−1/3, −*x*+*y*+1/3, −*z*+1/3; (iv) *x*−*y*+2/3, *x*+1/3, −*z*+1/3; (v) *y*+2/3, −*x*+*y*+4/3, −*z*+1/3; (vi) −*y*+2, *x*−*y*+1, *z*; (vii) −*x*+*y*+1, −*x*+2, *z*; (viii) −*x*+*y*+1, −*x*+1, *z*; (ix) −*y*+1, *x*−*y*, *z*; (x) *y*, −*x*+*y*, −*z*; (xi) *x*−*y*+1, *x*, −*z*.
